# Detection and Characterization of Metastatic Cancer Cells in the Mesogastrium of Gastric Cancer Patients

**DOI:** 10.1371/journal.pone.0142970

**Published:** 2015-11-13

**Authors:** Daxing Xie, Liang Liu, Hasan Osaiweran, Chaoran Yu, Fang Sheng, Chun Gao, Junbo Hu, Jianping Gong

**Affiliations:** 1 Tongji Cancer Research Institute, Tongji Hospital, Tongji Medical College in Huazhong University of Science and Technology, Wuhan, Hubei, China; 2 Department of Gastrointestinal Surgery, Tongji Hospital, Tongji Medical College in Huazhong University of Science and Technology, Wuhan, Hubei, China; University of Barcelona, SPAIN

## Abstract

Gastric cancer is the second leading cause of cancer death worldwide. Here, we propose a novel type of tumor metastasis designated as Metastasis V in gastric cancer. Metastasis V is defined as the appearance of cancer cells in the mesogastrium with perigastric adipose tissue. To detect its incidence and characterize its clinic pathological features, large cross sectional tissue analysis of mesogastrium from 74 patients were used. Metastasis V was detected in 1 of 40 (2.5%) patients with early gastric cancer, 8 of 34 (24%) patients with advanced gastric cancer. The mean distance of Metastasis V from gastric wall was approximately 2.6 cm. Metastasis V was closely associated with tumor invasion depth, along with a number of positive lymph node metastasis. The prognosis of patients with Metastasis V was significantly (*P*<0.05) worse than those with tumor cell-free mesogastrium. These findings indicate that by using whole-sectional analysis, Metastasis V can be detected in the mesogastrium of gastric cancer patients, and also suggests that it may be a risk factor for patient survival after radical surgery.

## Introduction

Although performed with curative intent and combined with neoadjuvant chemotherapy, radical surgery for advanced gastric cancer (Gastrectomy with D2 lymphadenectomy) is often followed by local-regional recurrence[1]. In fact, about 75–80% of cases still end up with local-regional recurrence in 2 years after the surgery[2]. Local-regional recurrence after curative radical gastrectomy is the main reason for poor prognosis of gastric cancer[3].

In the past three decades, TME (total mesorectal excision) or CME (complete mesocolic excision) surgical procedures for colorectal cancer have decreased the chances of local recurrence and increased 5-year survival [4,5]. Surgical research has suggested that the supposed reasons are better techniques, skilled surgeons with more years of practice, advanced peri-operation management, and complete lymph node dissections. On the other hand, it is still difficult to fully understand how patients who received R0 resection with staging of T2N0M0 and T3N0M0 also have local-regional recurrences, which can vary from 10% to 30%[6]. Thus, no exact causes or reason for it has been rigorously shown.

**Fig 1:**
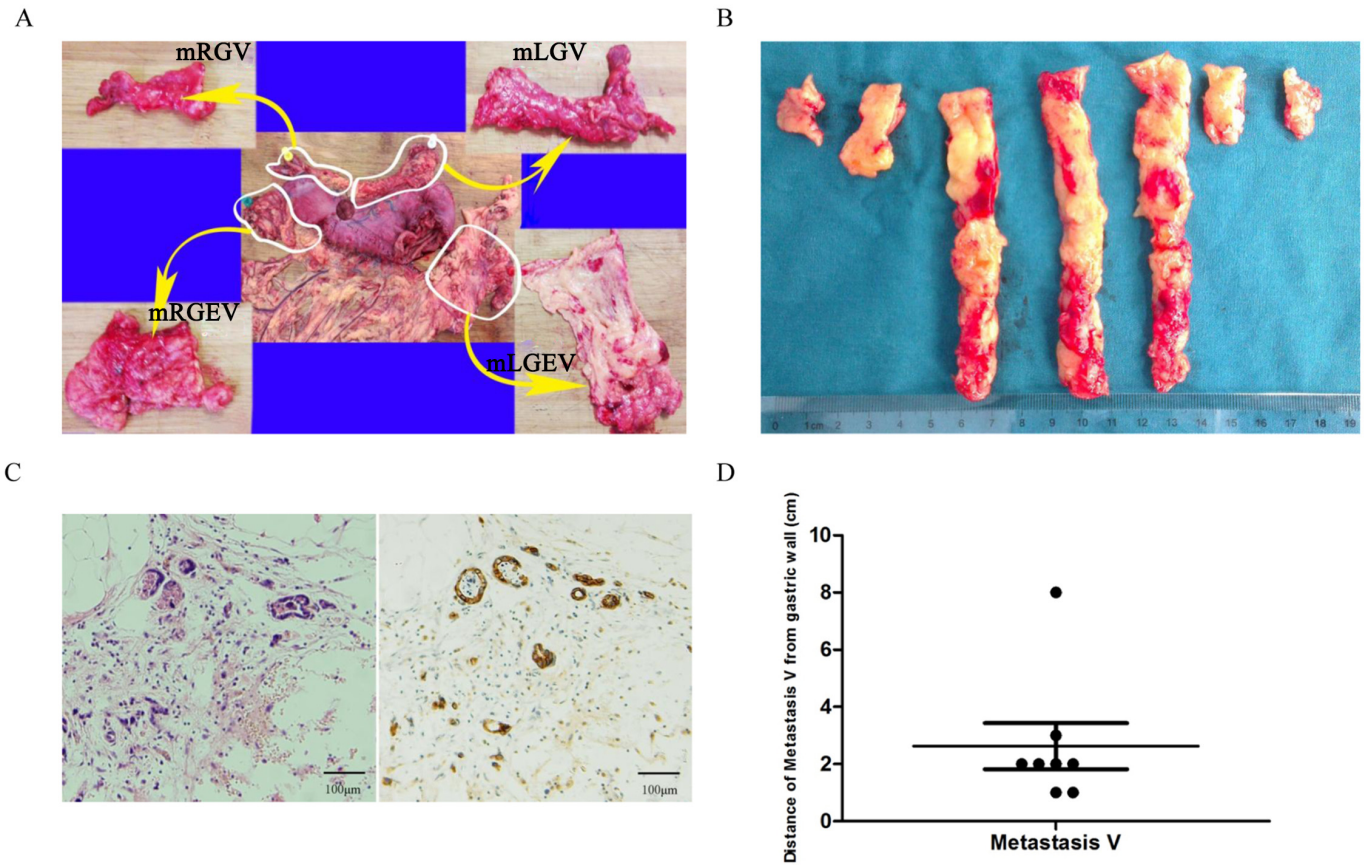
Detection of Metastasis V in gastric cancer patients. (A) Large cross sectional tissue samples analysis of mesogastrium from surgically resected specimens. mLGEV, mRGEV, mLGV and mRGV were analyzed. (B) Continuous sections at 1-cm-width intervals of mesogastrium specimens. (C) Isolated cancer cells were detected in the mesogastrium of resected gastric cancer specimens by both HE staining (left) and immunohistochemistry with CK AE1/AE3 antibody (right). (D) Distance of Metastasis V from the gastric walls.

Direct invasion, lymphatic drainage, hematogenous spread and peritoneal dissemination are the four classical routes through which local-regional recurrence or distant implants of gastric cancer cells can be determined. Each of these pathways is located in the particular cavity. For example, the direct invasion and peritoneal spread are located in the serous cavity; hematogenous metastasis can be found in blood vessel cavities; in lymphatic metastasis, the cancer cells are located in the cavities of lymphatic vessels and nodes. However, in addition to these pathways, people have also reported different metastatic cancer cells in local perigastric adipose tissues[7–11]. Since they failed to describe the cavity these tissues were located in, metastasis has been explained as a chance occurrence, occasional event, or one confused with an underlying serous spread. The “envelop” hypothesis of mesogastrium was proposed when a previously unidentified cavity, surrounded or enveloped by proper fascia, was discovered with cancer cells located and moving in it[12]. We designated the appearance of these cancer cells in the mesogastrium with perigastric adipose tissue as Metastasis V[12]. To confirm our hypothesis, we further detected the incidence and characterized the pathological characteristics of Metastasis V by using large cross sectional tissue samples of mesogastrium obtained from gastric cancer patients. A total of 5,892 mesogastrium samples sections, each with a thickness of 4 μm, from 74 patients were analyzed (Fig 1A and 1B).

## Materials and Methods

### Patients, specimens, and the large cross sectional tissue sample analysis

Seventy-four patients who underwent surgery for primary gastric cancer at the Department of Gastrointestinal Surgery, Tongji Hospital, Tongji Medical College in Huazhong University of Science and Technology between October 2012 and January 2014 were included in this study. The patients consisted of forty-two male and thirty-two female patients with a mean age of 52 (range, 23–72 years). All of these patients underwent laparoscopy assisted D2+CME (complete mesogastrium excision) radical gastrectomy with a curative R0 resection, and all the operation was performed by Prof. Jianping Gong, chief of GI surgery of Tongji Hospital, Huazhong University of Science and Technology. All participants provided informed written consent to participate in the study. This study was approved by the Tongji Hospital Ethics Committee.

The mesogastrium specimens were obtained from resected gastric tissues. In this study, four regions of the mesogastrium were included, and each named mesogastrium of Left Gastroepiploic Vessels (mLGEV), mesogastrium of Right Gastroepiploic Vessels (mRGEV), meosgastrium of Right Gastric Vessels (mRGV) and mesogastrium of Left Gastric Vessels (mLGV) respectively according to the main supplying vessels (Fig 1A). All resected specimens were fixed in 10% neutral-buffered formalin for at least one hour before any manipulation of the mesogastrium. The specimens were then straightened to identify the respective pedicles of the mesogastrium, and the sites of the primary tumor (upper (U), middle (M), or lower (L) parts, anterior or posterior walls, lesser or greater curvatures). The nearest distance between the primary tumor and each mesentery was measured and recorded (Fig 1A). The mesogastriums were extracted en bloc from the stomach, straightened and measured for their length and width. Specimens were cut perpendicular to the stomach wall at 1cm intervals and organized in a proximal to distal pattern marked by Roman numerals (Fig 1B). Each strip was cut into cubes parallel to the stomach wall at 1cm intervals. The cubes were then sequenced in radial patterns marked and numbered using standard numerals. The cubes were put into embedding cassettes, stored in 10% neutral-buffered formalin and transferred to the histopathology lab for further processing and large serial sectioning. A total of 5,892 sections, each 4 μm-thick, were removed from the 74 patients, with a mean of 80 blocks (range, 13–136) per patient. All of blocks were subjected to hematoxylin–eosin staining and immunohistochemistry. Table 1 shows the clinical and pathological characteristics of the 74 patients in our study. Clinicopathological data were analyzed according to the 7th Edition of the AJCC Cancer Staging Manual.

**Table 1:** Clinicopathological data of 74 patients.

Parameters	Results
Age (year, mean, range)	52(23–72)
Tumor size (cm) (mean±SD)	2.7±1.2
Sex (number of patients)	
Male	42
Female	32
Tumor location	
Upper	8
Middle	11
Lower	55
Histological grade	
Well	9
Moderate	30
Poor	35
Depth of invasion	
T1	40
T2	10
T3	20
T4	4
Lymph node metastasis	
N0	45
N1	18
N2	6
N3	5

Upper: upper third of the stomach; Middle: middle third of the stomach; Lower: lower third of the stomach. T1: invasion to lamina propria, muscularis mucosae, or submucosa; T2: invasion to muscularis propria; T3: invasion to subserosal connective tissue without invasion of visceral peritoneum or adjacent structures; T4: invasion to serosa (visceral peritoneum) or adjacent structures. N0: no regional lymph node metastasis; N1: metastasis in 1 to 2 regional lymph nodes; N2: metastasis in 3 to 6 regional lymph nodes; N3: metastasis in 7 or more regional lymph nodes. T and N categories were based upon the 7th Edition of the AJCC Cancer Staging Manual.

For survival analysis, a total of sixty-seven patients who received laparoscopic D2 gastrectomy plus complete mesogastrium excision with a curative R0 resection between February 2009 to June 2012 were included. All the operation was performed by Prof. Jianping Gong. After the surgical resection, all patients underwent a follow-up, with the median follow-up at analysis being 47 months for all patients. The follow-up programme of post-operative surveillance consisted of physical examination, blood chemistry including CEA, computed tomography, and ultrasound performed every 3 months to diagnose recurrent diseases.

### Immunohistochemistry and definition of Metastasis V in mesogastrium

Immunohistochemistry was performed using the cytokeratin AE1/AE3 antibody, a monoclonal antibody that reacts to epithelial tumor cells from gastrointestinal organs and has been proven to be a highly sensitive marker for gastric cancers.

Immunostaining was performed according to the standard streptavidin-biotin method. Briefly, the sections were deparaffinized and rehydrated, and endogenous peroxidase was inhibited with 0.3% H_2_O_2_. For antigen retrieval, sections were boiled in 0.01M, pH 6.0 sodium citrate buffer for 15 min in a microwave oven. After blocking with 5% normal goat serum for 30 min, the primary anti-cytokeratin AE1/AE3 monoclonal antibody (1:100) was now in blocking buffers and the sections were incubated at 4°C overnight. Peroxidase/DAB, rabbit/Mouse, DAKO from EnVision™ Detection Systems were applied in the following steps. The sections were counterstained with Mayer’s hematoxylin, dehydrated in a graded alcohol series, cleared in xylene, and mounted. Metastasis V is defined as the presence of isolated cancer cells in the mesogastrium that is discontinuous from the primary lesion and no lymph nodes detected on the same slide, as detected by HE staining and immunostaining for cytokeratin AE1/AE3.

### Immunohistochemistry image analysis

The intensity of the reaction product of E-cadherin and DAB2IP immunohistochemistry was measured semi-quantitatively using Image Pro Plus Software 6.0 (Media Cybernetics, CA, USA). This procedure can be divided into seven different steps: 1. creating and measuring the Area of Interest (AOI); 2. calibrating the optical density; 3. acquiring, converting and saving images; 4. performing the background and background staining correction; 5.setting of the AOI in the acquired image to measure the optical density; 6.measuring density; 7.creating macros[13].

### Statistical Analysis

Statistical analyses were performed by SPSS version 17.0. Descriptive data were presented as mean±SD. The X^2^ test, Fisher’s exact test were be used to determine the significance of the differences between the covariates. The survival durations were calculated using the Kaplan–Meier method and were analyzed by the log–rank test to compare the cumulative survival durations in the patient groups. The survival curve was calculated from the date of surgery. The Cox proportional hazards model was used to compute the univariate and multivariate hazards ratios for the study parameters. For all of the tests, a P-value of less than 0.05 was considered to be statistically significant.

## Results and Discussion

### Detection of Metastasis V

Metastasis V was detected in 9 of total 74 (12%) patients by H&E staining and immunostaining (Fig 1C), including 1 of 40 (2.5%) patients with early gastric cancer, 8 of 34 (24%) patients with advanced gastric cancer (Table 2). These isolated cancer cells are discontinuous from the primary lesion and no lymph nodes was observed in the same slide (Fig 1C). The clinic pathological features of Metastasis V positive patients were shown in Table 2. Of these patients, five had poorly differentiated adenocarcinoma and four had moderately differentiated adenocarcinoma. In terms of depth of tumor invasion, two had tumor penetration of the serosa, six had a sub-serosal invasion and one had a sub-mucosal penetration (Table 2). Although eight Metastasis V-positive patients had positive lymph node metastasis, one Metastasis V-positive patient without lymph node metastasis was also identified.

**Table 2:** Clinicopathological data of patients with Metastasis V.

Patients	Age/ gender	Tumor size(cm)	Tumor location	Metastasis V location	Histological grade	Depth of Invasion	Lymph node metastasis	TNM Staging
1	44/Female	2.0	Lower	mRGEV	Por	Serosal	Positive(7/26)	T3N3M0
2	34/Female	3.0	Upper	mLGV	Por	Serosal	Positive(1/21)	T3N1M0
3	54/Male	3.0	Upper	mLGV	Por	Serosal	Positive(4/22)	T3N1M0
4	54/Male	5.0	Lower	mRGEV	Mod	Submucosa	Positive(8/30)	T1bN3M0
5	61/Male	2.0	Lower	mLGV, mRGV	Mod	Serosal	Positive(3/34)	T3N2M0
6	66/Male	5.0	Middle	mLGV	Por	Serosal	Positive(2/29)	T4aN1M0
7	69/Male	1.5	Middle	mLGV	Por	Serosal	Positive(2/13)	T4aN1M0
8	38/Female	3.0	Lower	mRGEV	Mod	Serosal	Positive(22/32)	T3N3M0
9	71/Male	3.0	Lower	mRGEV	Mod	Subserosal	Negative(0/28)	T3N0M0

Por: poorly differentiated adenocarcinoma, Mod: moderately differentiated adenocarcinoma.

### Distribution and distance of Metastasis V

**Fig 2:**
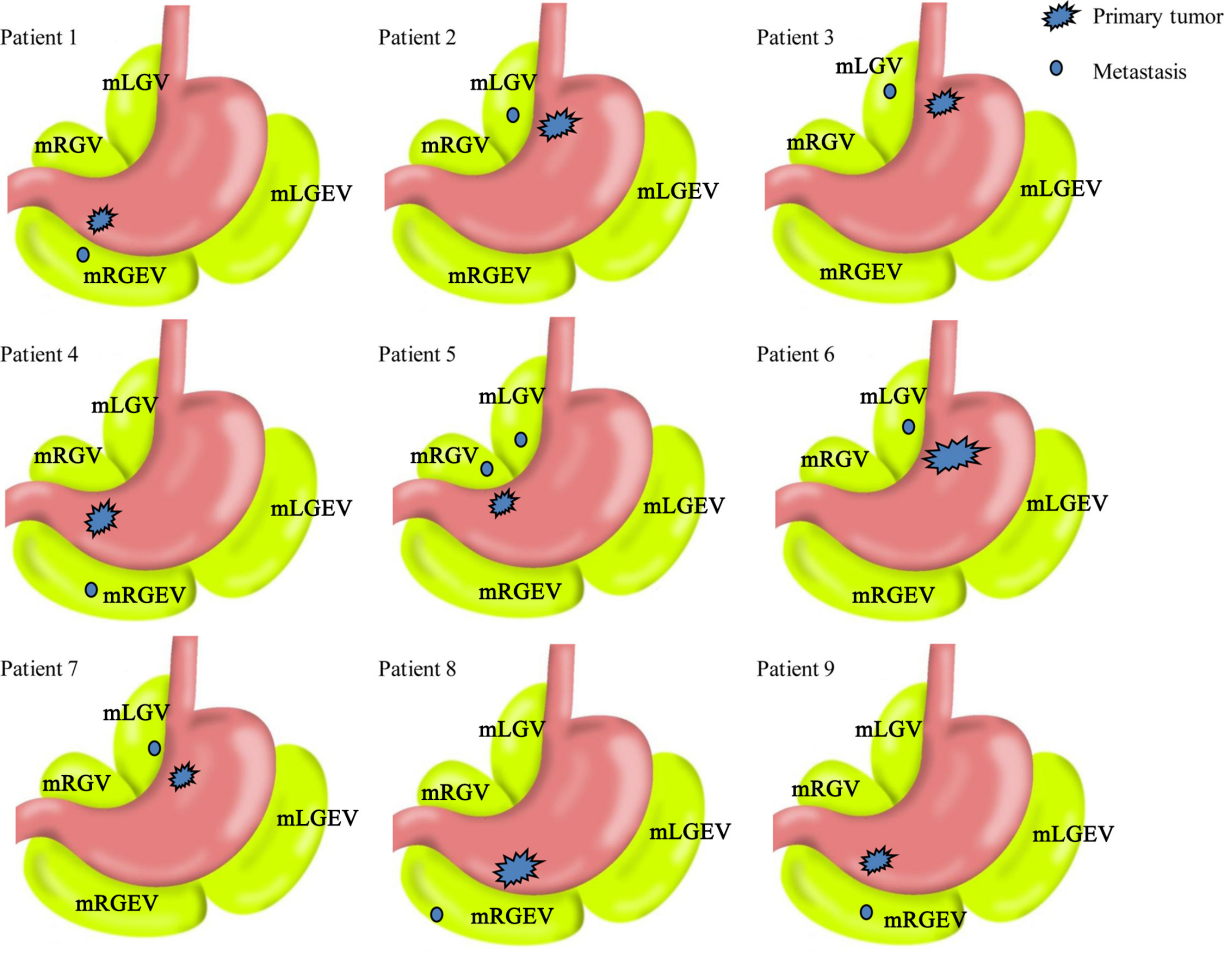
Schema of locational relationship between primary tumor and Metastasis V in nine patients with Metastasis V.

To identify the distribution of Metastasis V, we analyzed the relationships between localization of Metastasis V and the primary tumor lesions, as well as the distance of Metastasis V from the gastric walls. Of these nine Metastasis V-positive patients, the location of Metastasis V with respect to the primary tumor in five patients were detected in the mLGV close to the left gastric artery (lymph node No. 7 station), the common hepatic artery (lymph node No. 8 station) and the celiac trunk (lymph node No. 9 station) (Fig 2). The remaining fours were found in the mRGEV close to the infropyloric lymph nodes (No. 6 station) (Fig 2). Interestingly, lesions of Metastasis V were found in both mLGV and mRGV close to the suprapyloric lymph nodes (No. 5 station) in one patient (Patient 5 of Fig 2).

The mean distance of Metastasis V from gastric wall was approximate 2.6 cm (range, 1–8 cm) (Fig 1D). The range of distances of Metastasis V from the gastric walls was 1–3 cm in 8 out of the 9 patients expect one patient (Patient 8) in which the distance was 8 cm. This may be due to the mesogastrium in abdominal cavity lengthening out and changing from a three-dimensional form when resected by the surgeon.

### Correlation between Metastasis V and clinical factors

The demographic characteristics of patients and the pathologic features of tumors with Metastasis V, both positives and negatives, are shown in Table 3. The incidence of Metastasis V was closely related to the depth of the primary tumor invasion. Metastasis V was more frequent in tumors invading the subserosal (6/20 tumors; 30%) or serosal layer (2/4 tumors; 50%) compared with tumors that invading the submucosa layer (1/40 tumors; 2.5%), which was statistically significant (P = 0.001). Moreover, lymph node metastasis was also closely associated with Metastasis V. Metastasis V was more frequent as the number of lymph node metastasis increased (N0:1/45, 2.2%; N1:4/18, 22.2%; N2:1/6, 16.7%; N3:3/5, 60%; respectively, P = 0.001). Poorly differentiated tumors were more likely to have Metastasis V, but is not statistically significant (P = 0.489). There were no significant differences between Metastasis V-positives and Metastasis V-negatives in terms of sex, age, tumor size, tumor location and histological subtypes (Table 3 and S1 Table).

**Table 3:** Correlation between Metastasis V and clinicopathologic findings (N = 74).

Variables	Metastasis V		P value ^1^
	Positive (n = 9)	Negative (n = 65)	
Sex			
Male	6	36	NS
Female	3	29	
Tumor size (cm)			
2.7±1.2 (mean±SD)	3.1±1.2	2.6±1.2	NS
Age (year)			
Average (52±11)	54±14	48±11	NS
Tumor location			
Upper	2	6	NS
Middle	2	9	
Lower	5	50	
Histological grade			
Well	0	9	NS
Moderate	4	26	
Poor	5	30	
Depth of invasion			
T1	1	39	0.001
T2	0	10	
T3	6	14	
T4	2	2	
Lymph node metastasis			
N0	1	44	0.001
N1	4	14	
N2	1	5	
N3	3	2	

1. *X* ^2^ test or Fisher’s exact test

### Survival

**Fig 3:**
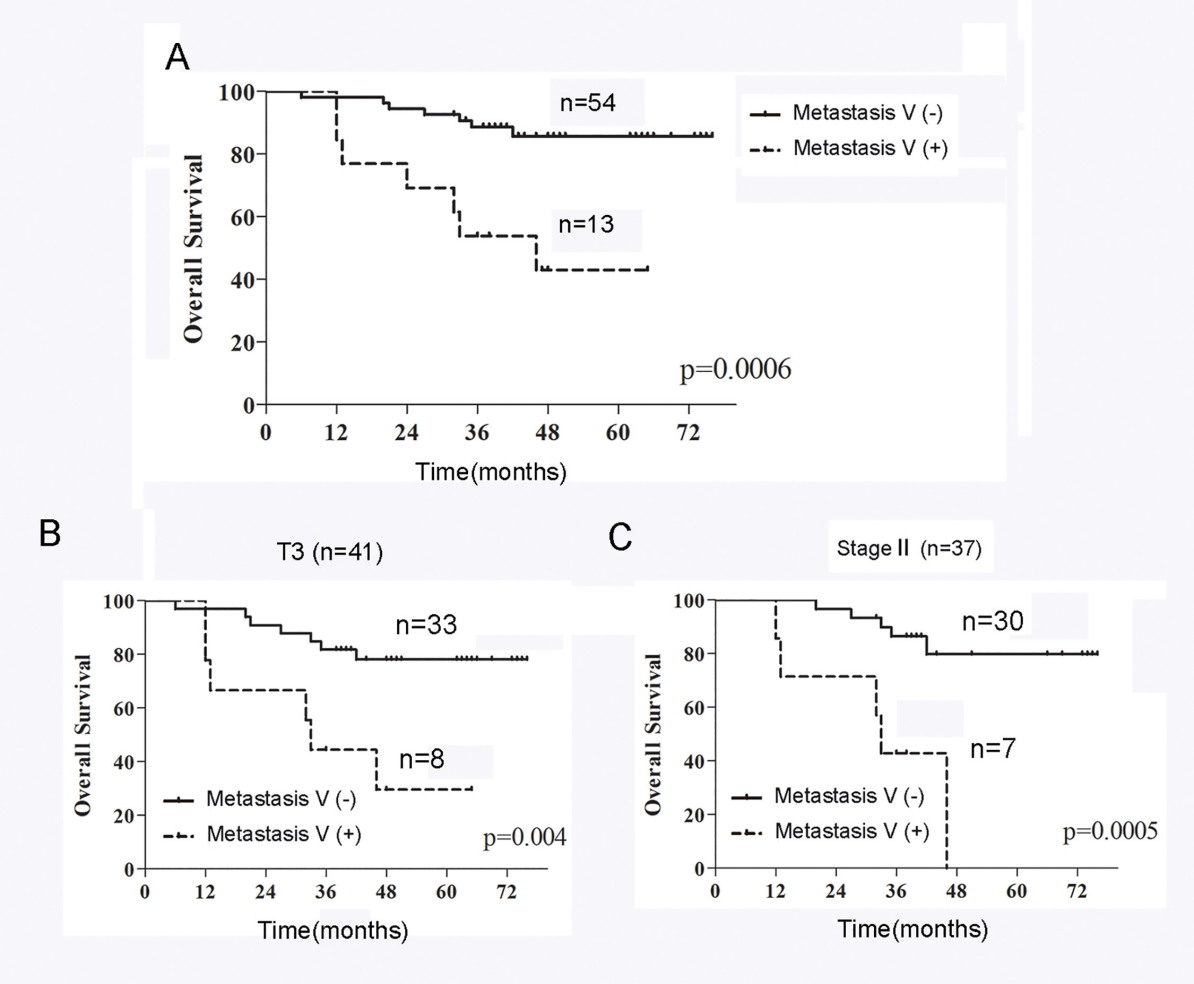
The overall survival of patients with or without Metastasis V. (A) The Kaplan–Meier overall survival curves showed the prognosis of Metastasis V-positive patients was significantly (P = 0.006) worse than Metastasis V-negative patients. (B-C) Metastasis V-positive patients had a significantly poorer prognosis than Metastasis V-negative patients in the T3 subgroup (P = 0.004; B) or in clinical stage III (P = 0.0005; C).

Based on our follow-up data, the prognosis of all Metastasis V-positive patients was significantly (P = 0.006) worse than Metastasis V-negative patients (Fig 3A). In addition, we analysed the prognostic significance of Metastasis V in the different tumor subgroups (T2, T3 and T4) or clinical stages (II and III). The prognosis of the patients with Metastasis V-positive was significantly poorer than those with Metastasis V-negative in the T3 subgroup (P = 0.004; Fig 3B) or in clinical stage II (P = 0.0005; Fig 3C), while no significant difference in prognosis was found between Metastasis V-positives and Metastasis V-negatives in the T2, T4 subgroups or in stage III (data not shown). We also evaluated prognostic factors affecting overall survival. According to a univariate analysis, the size diameter (P = 0.02), Laurén classification (P = 0.01) and Metastasis V (P = 0.002) were significantly correlated with patient survival (S2 Table). In a multivariate analysis, Metastasis V (P = 0.037) was found to be independent prognostic factors (S3 Table).

### DAB2IP and E-cadherin are down-regulated in Metastasis V

**Fig 4:**
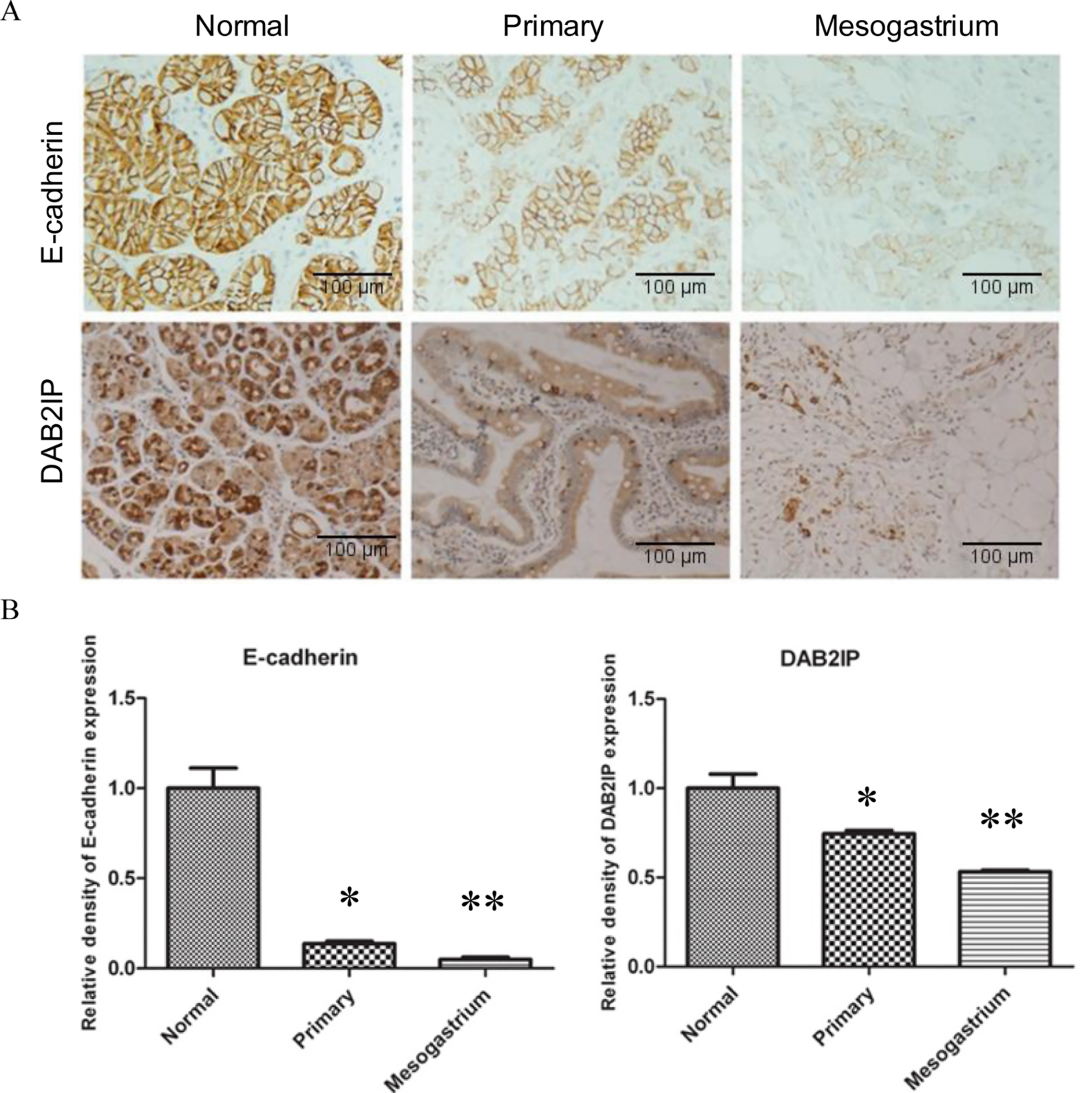
E-cadherin and DAB2IP expression in normal gastric mucosa, primary gastric tumors and Metastasis V within the mesogastrium. (A) Representative IHC staining for DAB2IP and E-cadherin from the same patient. (B) The relative quantitative analysis of E-cadherin and DAB2IP expression. One asterisk indicated statistical significance in normal mucosa vs. primary tumors (*, P < 0.01). Two asterisks indicated statistical significance in primary tumors vs. mesogastrium (**, P<0.01).

In order to evaluate the clinical significance of the results and develop potential therapeutic target for Metastasis V, it was necessary to investigate the underlying molecular mechanisms. E-cadherin is regarded as a major marker for EMT (epithelial-to-mesenchymal transition), known as a critical process in the biology of cancer metastasis[14]. Our previous studies demonstrated that the loss of DAB2IP expression initiated EMT and promoted tumor invasion and metastasis[15]. In this study, the mesogastrium metastasis and its matching primary tumor and adjacent normal tissue were immunostained for DAB2IP and E-cadherin. Our results showed that the expression of both DAB2IP and E-cadherin decreased in the mesogastrium metastasis compared with that in the matched primary tumor and the adjacent normal tissue (Fig 4), which suggests that DAB2IP-regulated EMT may play a role in Metastasis V. Since the downstream of DAB2IP-mediated Wnt pathway is the activation of transcriptional factors such as ß-catenin and p65, we also detected the expression of ß-catenin and p65. Our results showed that the expression of both ß-catenin and p65 increased (as well as nuclear localization) in the mesogastrium metastasis compared with that in primary tumor (S1 Fig). The exact regulatory mechanisms, however, need to be further investigated.

## Discussion

In our study, it is clear that metastatic cancer cells reside in the adipose tissues around the stomach of gastric cancer patient. These metastatic gastric cancer cells showed three primary characteristics: (1). they are located in adipose tissues and separated from the primary tumor; (2). there were no structures of blood vessel, lymphatic nodes or other vessels around these metastatic cells (Fig 1C); (3). the adipose tissues were enveloped by proper fascia. These characteristics demonstrated that this type of metastasis was different from the four classic types of metastasis, including direct invasion, serous spread, lymphatic metastasis or hematogenous spread. Since it was a new pathway, we designated it, for the moment, as Metastasis V[12]. The metastatic cancer cells usually spread within different cavities by different forces, for example, hematogenous metastasis spreads in blood vessel cavities driven by circulation of the blood stream, lymph node metastasis spreads in the lymphatic cavities driven by circulation of the lymphatic stream, whilst direct infiltration and serous spreads are in the serous cavities which shows a free autonomous spread or movement. Our previous data suggested that Metastasis V was observed to be spreading within the proper fascia cavity, and the data from this study further suggests that DAB2IP-mediated EMT might be involved in Metastasis V spreading through fat tissues (Fig 4).

Metastasis V was closely correlated with tumor invasion depth in the stomach wall. Our data shows that most instances of Metastasis V occurred in T3 or T4a tumors, although some also occurred in T1 tumors (Table 2). Metastasis V was also correlated with lymphatic metastasis. Lymphatic nodes staging N1-N3 had larger percentages of Metastasis V than N0, and though N0 had instances of Metastasis V (Table 3). Moreover, the prognosis of the patients with Metastasis V-positive tumours was significantly worse than those with Metastasis V-negative tumours, especially in T3 or stage II (Fig 3). Due to the limited number of patients in current study, a larger sample of cohort patients should be enrolled for further analysis. These findings suggest that detection of Metastasis V has a poor prognostic relevance in patients who are undergoing a curative resection. Since D2 radical gastrectomy can only limitedly improve the survival of advanced gastric cancer patients, detection of Metastasis V within the mesogastrium resected should be an effective method for the selection of patients for further therapeutic chemotherapy or other target therapies. The exact route or mechanism of Metastasis V is not clear. It is assumed that when the primary tumor invades the gastric wall to a certain extent, tumor cells drop off from the primary site possible due to DAB2IP-regulated EMT mechanism, and then spread among the fat tissues within the proper fascia cavity of mesogastrium which lies between the stomach and mesentery[12].

Metastasis V is not limited only to gastric cancer but can also exist in many other cancers originating from various tissues, including rectal cancer, nephrogenic adenoma, et al[16,17]. In our study, there was an incidence of 24% of Metastasis V in advanced gastric cancer, and such percentage can be expected to increase when larger cross sections of one slide per 0.5 or 0.25 cm are made.

Although others have shown, through large serial sections of mesogastrium of greater and lesser curvatures, that the incidence of tumor nodules in mesogastrium can be as high as 8%[11], the pathological features and incidence of Metastasis V are not fully studied. Firstly, based on embryonic anatomy, the greater omentum and the lesser omentum are not fully mesogastrium. Mesogastrium should possess two characteristics: one is that it should be located along the edge of stomach, the other is that it should enclose the main blood vessels of mesentery that connect them to attached organs. In our study, four regions of the mesogastrium were resected by laparoscopy assisted D2+CME (complete mesogastrium excision) radical gastrectomy, and each region was named mLGEV, mRGEV, mLGV, mRGV respectively (Fig 1A). We further analyzed the localization of Metastasis V within different regions of mesogastrium. Our data showed that mLGV and mRGEV are more frequently detected with Metastasis V than other mesogastrium (Fig 2), which implies the clinic significance of these two parts during radical gastrectomy. Further study on the distance of Metastasis V from the gastric walls suggested the range or length of mesogastrium to be resected during radical surgery (Fig 1D).

It has been well known in practice that radical surgery for cancers should include en bloc resections of the primary tumor and neighboring tissues. However, it is difficult to understand the exact boundaries of en bloc resection leading to a more complete lymphadenectomy being undertaken. The model of Metastasis V within mesogastrium which is enveloped by the proper fascia proposes a precise boundary and pathologic direction which can not be covered by the model of lymphatic metastasis. Moreover, our findings are clinically significant because metastasis through this pathway invariably requires surgical excision as the treatment of choice. Given that local-regional recurrence might be closely associated with Metastasis V, complete mesogastrium excision (CME) should be accomplished along with gastrectomy and D2 lymph node dissection to reduce the incidence of local-regional recurrence in gastric cancer[18]. A randomized control trial (NCT01978444) is currently underway in our department to evaluate the clinical significance of surgical excision of Metastasis V.

## Supporting Information

S1 Table Correlation between Metastasis V and histological subtypes (N = 74). (DOCX)

S2 Table Univariate analysis for factors affecting overall survival in 67 patients. (DOCX)

S3 Table Multivariate analysis for factors affecting overall survival in 67 patients. (DOCX)

S1 Fig Expression of p65 and β-catenin in normal gastric mucosa, primary gastric tumors and Metastasis V within the mesogastrium. Representative IHC staining for p65 and β-catenin from the same patient.
(TIF)
