# Correction: Detection and Characterization of Metastatic Cancer Cells in the Mesogastrium of Gastric Cancer Patients

**DOI:** 10.1371/journal.pone.0148681

**Published:** 2016-01-29

**Authors:** The PLOS ONE Staff

Daxing Xie is incorrectly designated as the corresponding author. The correct corresponding author is Jianping Gong. Jianping Gong’s email address is: jpgong@tjh.tjmu.edu.cn. The publisher apologizes for the error.
