# Supplementary material for: Detection and Characterization of Metastatic Cancer Cells in the Mesogastrium of Gastric Cancer Patients
Source: PLoS One. 2015 Nov 13;10(11):e0142970. doi: 10.1371/journal.pone.0142970 (PMC4643961; doi:10.1371/journal.pone.0142970)
Supplement: S1 Table — (DOCX) [file pone.0142970.s001.docx]

| Laurén classification | Metastasis V | | P value^1^ |
| --- | --- | --- | --- |
|  | Positive(n=9) | Negative(n=65) |  |
| Diffuse | 2 | 20 | NS |
| Intestinal | 6 | 40 |  |
| Mix | 1 | 5 |  |

**S1 Table. Correlation between Metastasis V and histological subtypes (N= 74).**
