# Supplementary material for: Detection and Characterization of Metastatic Cancer Cells in the Mesogastrium of Gastric Cancer Patients
Source: PLoS One. 2015 Nov 13;10(11):e0142970. doi: 10.1371/journal.pone.0142970 (PMC4643961; doi:10.1371/journal.pone.0142970)
Supplement: S2 Table — (DOCX) [file pone.0142970.s002.docx]

| **Univariate analysis for factors affecting overall survival in 67 patients** | | | | |
| --- | --- | --- | --- | --- |
| Variables | No. | Mean (SEM)  Disease-free survival, mo | 3-y Survival  Rate,% | P Value |
| **Gender** |  |  |  |  |
| Male | 40 | 46.7 (18.1) | 82.5% | P=0.38 |
| Female | 27 | 41.0 (13.3) | 64.0% |  |
| **Age** |  |  |  |  |
| >60 | 16 | 45.1 (19.1) | 66.7% | P=0.41 |
| ≤60 | 51 | 44.1 (15.6) | 80.3% |  |
| **Size Diameter** |  |  |  |  |
| >5cm | 24 | 38.9 (16.2) | 58.2% | P=0.02 |
| ≤5cm | 43 | 48.3 (15.7) | 88.3% |  |
| **Location** |  |  |  |  |
| Upper | 16 | 45.8 (15.1) | 69.6% |  |
| Middle | 14 | 45.8 (18.8) | 78.6% | P=0.92 |
| Lower | 37 | 43.2 (16.5) | 77.6% |  |
| **Laurén classification** |  |  |  |  |
| Intestinal | 17 | 43.7 (17.9) | 82.3% |  |
| diffuse | 47 | 45.0 (16.5) | 82.8% | P=0.004 |
| mix | 3 | 36.0 (14.0) | 0% |  |
| **Differentiation** |  |  |  |  |
| Well | 2 | 43.5 (44.5) | 50.0% |  |
| Moderately | 9 | 43.8 (12.3) | 88.9% | P=0.35 |
| Poorly | 56 | 44.5 (16.3) | 75.6% |  |
| **T stage** |  |  |  |  |
| T1 | - | - | - | P=0.07 |
| T2 | 6 | 37.8 (4.0) | 100.0% |  |
| T3 | 42 | 47.5 (19.8) | 67.8% |  |
| T4 | 19 | 39.6 (5.8) | 94.7% |  |
| **N stage** |  |  |  |  |
| N0 | 11 | 39.1 (2.6) | 100.0% |  |
| N1 | 38 | 46.1 (17.8) | 69.9% | P=0.24 |
| N2 | 9 | 43.1 (11.8) | 88.9% |  |
| N3 | 9 | 44.8 (24.6) | 66.7% |  |
| **Metastasis V** |  |  |  |  |
| Positive | 13 | 34.8 (16.2) | 43.1% | P<0.01 |
| Negative | 54 | 46.7 (15.8) | 85.7% |  |
| **Adjuvant chemo- treatment** |  |  |  |  |
| Received | 38 | 42.9 (16.7) | 71.3% | P=0.21 |
| Non | 29 | 46.3 (16.2) | 8.28% |  |

**S2 Table. Univariate analysis for the factors affecting overall survival in 67 patients.**
