# Supplementary material for: Detection and Characterization of Metastatic Cancer Cells in the Mesogastrium of Gastric Cancer Patients
Source: PLoS One. 2015 Nov 13;10(11):e0142970. doi: 10.1371/journal.pone.0142970 (PMC4643961; doi:10.1371/journal.pone.0142970)
Supplement: S3 Table — (DOCX) [file pone.0142970.s003.docx]

| **Multivariate analysis for factors affecting overall survival in 67 patients** | | | |
| --- | --- | --- | --- |
| **Factors** | P value | Odds ratio | 95% CI |
| Size Diameter | 0.535 | 1.551 | 0.388-6.198 |
| Laurén classification | 0.297 | 1.768 | 0.605-5.161 |
| Metastasis V | 0.021 | 4.155 | 1.244-13.882 |

**S3 Table. Multivariate analysis for factors affecting overall survival.**
