# Supplementary figures and images for: Detection and Characterization of Metastatic Cancer Cells in the Mesogastrium of Gastric Cancer Patients
Source: PLoS One. 2015 Nov 13;10(11):e0142970. doi: 10.1371/journal.pone.0142970 (PMC4643961; doi:10.1371/journal.pone.0142970)

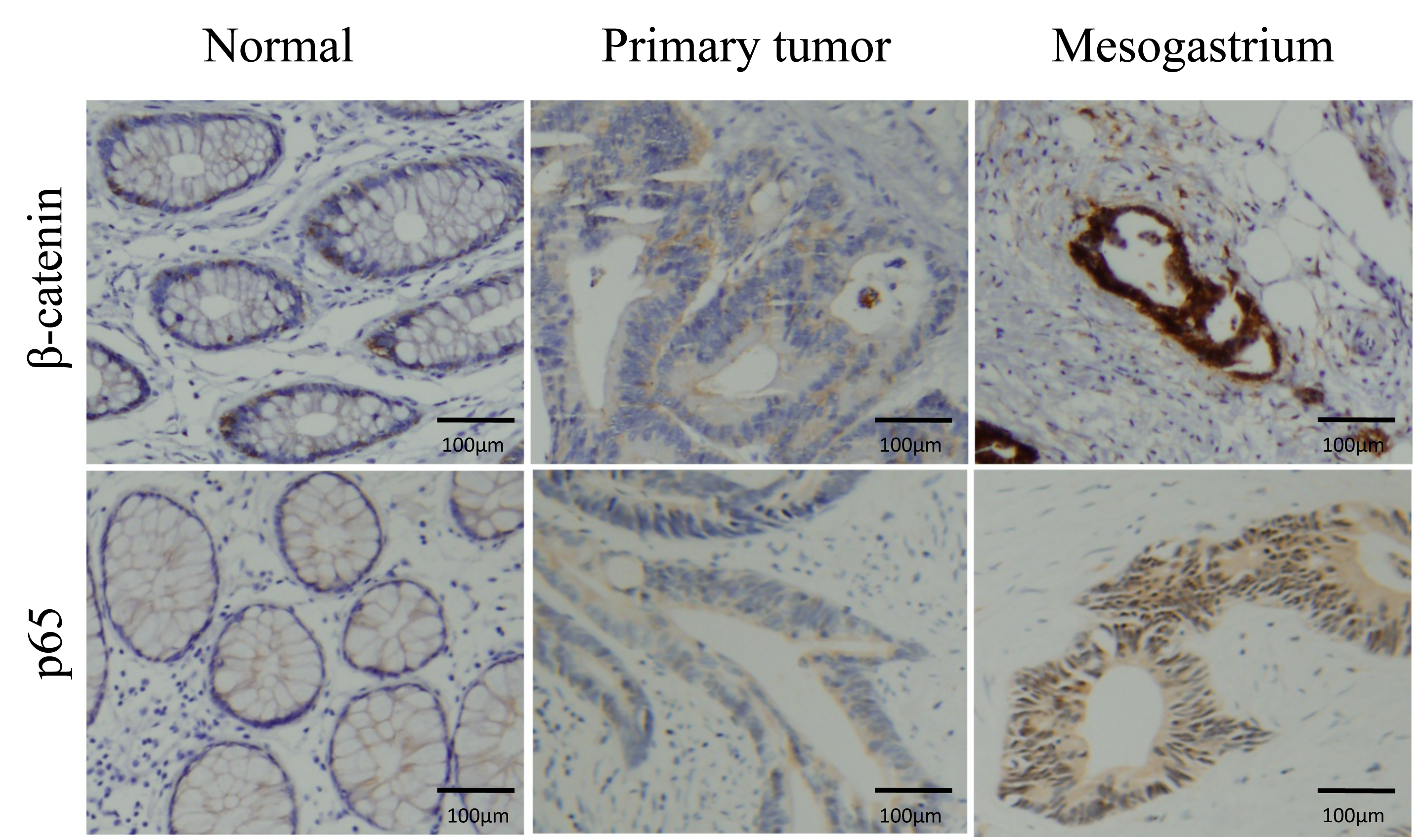

Supplement: S1 Fig — Representative IHC staining for p65 and β-catenin from the same patient. (TIF) [file pone.0142970.s004.tif]
